# Real-World Experience, Effectiveness, and Safety of Direct-Acting Antivirals for the Treatment of Hepatitis C in Oman: A Cross-Sectional, Multicenter Study

**DOI:** 10.3390/jcm13237411

**Published:** 2024-12-05

**Authors:** Khalid M. Al-Naamani, Heba Omar, Said A. Al Busafi, Halima H. Al Shuaili, Zakariya Al-Naamani, Murtadha Al-Khabori, Elias A. Said, Abdullah H. AlKalbani, B. R. Kamath, Bashar Emad, Shahina Daar, Lolo Alhajri, Alya AlKalbani, Zainab AlFarsi, Haifa Alzuhaibi

**Affiliations:** 1 Department of Internal Medicine, Division of Gastroenterology & Hepatology, The Medical City of Military and Security Services, Muscat 111, Oman; hebaomar1202@hotmail.com (H.O.); halima.alshueili@gmail.com (H.H.A.S.); ahs92@hotmail.com (A.H.A.); brkamat@gmail.com (B.R.K.); 2 Endemic Medicine and Hepatology Department, Faculty of Medicine, Cairo University, Cairo 11652, Egypt; 3 Department of Medicine, College of Medicine and Health Sciences, Sultan Qaboos University, Muscat 123, Oman; busafis@squ.edu.om (S.A.A.B.); z.alnaamani@squ.edu.om (Z.A.-N.); 4 Department of Hematology, College of Medicine and Health Sciences, Sultan Qaboos University, Muscat 123, Oman; khabori@squ.edu.om (M.A.-K.); sf.daar@gmail.com (S.D.); 5 Department of Microbiology and Immunology, College of Medicine and Health Sciences, Sultan Qaboos University, Muscat 123, Oman; esaid@squ.edu.om; 6 Department of Medicine, Jordan University of Science and Technology, Ar-Ramtha 22110, Jordan; beabumallouh19@med.just.edu.jo; 7 Department of Nursing, The Medical City of Military and Security Services, Muscat 111, Oman; nifty_icno@hotmail.com (L.A.); alya20111@gmail.com (A.A.); 8 Department of Nursing, Sultan Qaboos University Hospital, University Medical City, Muscat 123, Oman; zainabh@squ.edu.om (Z.A.); haifaz@squ.edu.om (H.A.)

**Keywords:** hepatitis C, direct-acting antiviral agents, sofosbuvir, daclatasvir, velpatasvir, treatment efficacy, sustained virologic response, safety, adverse drug effects, Oman

## Abstract

**Background**: The advent of direct-acting antiviral (DAA) therapy has revolutionized the treatment landscape of the hepatitis C virus (HCV) infection. This study aimed to provide a comprehensive research study of the real-world effectiveness and safety of DAA treatment, representing the first study conducted in the Omani population. **Methods**: A cross-sectional study was conducted including 375 HCV patients with different genotypes, treated using different DAA regimens, with or without ribavirin, between January 2012 and December 2020 at the Sultan Qaboos University Hospital and the medical city for military and security services, two tertiary hospitals in Muscat, Oman. The rate of sustained virologic response 12 weeks after completing the regimen (SVR-12) was analyzed as the primary outcome. Secondary outcomes included treatment safety and adverse events related to DAA therapy, as reported by patients and treating physicians. **Results**: A total of 375 patients were included in the study, with a mean age of 47.3 ± 15.4 years. Most were male (59.2%) and treatment-naïve (71.7%). The prevalence of liver cirrhosis was 19.7%, while 4.0% had hepatocellular carcinoma (HCC). The SVR-12 rate among treatment-naïve and treatment-experienced patients was 95.0% and 93.4%, respectively. Several parameters were associated with DAA treatment failure, including liver cirrhosis (*p* = 0.004) and active HCC (*p* = 0.009). Following SVR-12, significant improvements were observed in alanine transaminase, bilirubin, and albumin levels, Fibrosis-4 Index, and liver stiffness measurements compared to baseline (*p* <0.001 each). No significant adverse effects were reported. **Conclusions**: Based on our real-world experience, DAAs are highly effective in treating patients with HCV infection in Oman, with an excellent tolerability and safety profile.

## 1. Introduction

The World Health Organization has set a target for the elimination of the hepatitis C virus (HCV) by 2030 [1]. Currently, an estimated 50 million individuals globally live with chronic HCV infection, with approximately 242,000 deaths occurring each year due to HCV-related complications, including cirrhosis and hepatocellular carcinoma [1]. Importantly, HCV infection is one of the leading causes of liver cirrhosis and hepatocellular carcinoma (HCC) and is ranked as the foremost indication for liver transplantation worldwide [1,2,3]. Chronic HCV causes chronic inflammation, oxidative stress, and direct viral effects on hepatocyte proliferation and apoptosis, leading to fibrosis and HCC [2].

However, since the introduction of direct-acting antiviral (DAA) therapies, the treatment landscape of HCV has changed markedly due to the high efficacy rate and favorable safety profile of these medications, enabling sustained virologic response (SVR) even in difficult-to-treat patient populations such as those with decompensated cirrhosis [4]. Several prior studies have delineated associations between achieving SVR and reductions in liver-related progression and mortality, particularly in patients without liver cirrhosis or those with compensated liver cirrhosis [5,6,7,8].

Typically, different DAA regimens are recommended according to specific HCV genotypes; however, certain regimens―such as sofosbuvir (SOF)/velpatasvir (VEL) and glecaprevir (GLE)/pibrentasvir (PIB)―are pan-genotypic and have been to show to result in high SVR in difficult-to-treat subpopulations [9,10]. Previously, the high cost of proprietary DAAs hindered their widespread use in many countries [11]. However, the availability of generic formulations has enabled treatment for millions of people around the world, with these versions demonstrating comparable efficacy and safety profiles [12,13]. Nonetheless, the randomized controlled trials mandated for regulatory approval may not entirely capture the efficacy rates and side-effect profiles of medications in clinical practice, as such trials often employ stringent inclusion criteria. Consequently, real-world evidence is essential to supplement findings from regulatory trials [14]. The widespread use of various DAA regimens across the world over the last 13 years thus represents a valuable resource for evaluating the efficacy and safety of these drugs in diverse patient populations.

In Oman, the estimated prevalence of individuals with antibodies against HCV (anti-HCV) is approximately 0.5%, with a viremic prevalence of 0.4% [15]. This incidence is higher among high-risk groups; a previous study assessing HCV markers in 200 multi-transfused thalassemia patients found that 41% were positive for anti-HCV [16]. However, the efficacy of treatment using pegylated interferon and ribavirin (RBV)―which was the standard treatment before the advent of DAAs―in such groups is low, and the side-effects profile is high [17]. Instead, the utilization of DAAs in this population has resulted in high rates of SVR with minimal side effects [18]. Various SOF-based regimens, particularly in combination with ledipasvir (LDV), daclatasvir (DCV), or VEL, have been introduced to treat HCV infections in Oman. To date, no published studies have specifically evaluated the effectiveness of DAAs in Omani patients, highlighting a critical gap in understanding the regional variability in treatment outcomes. The primary objective of the present study was to present our real-world experience with different DAA regimens across diverse HCV patient populations in Oman, including those traditionally considered challenging to treat, while concurrently assessing their efficacy and safety profiles.

## 2. Materials and Methods

### 2.1. Study Design, Setting, and Population

This cross-sectional study was conducted between January 2012 and December 2020 at the Medical City for Military and Security Services (MCMSS) and Sultan Qaboos University Hospital (SQUH), two tertiary hospitals in Muscat, the capital city of Oman. Both of these hospitals houses specialized hepatology clinics to which most HCV cases from other regions in Oman are referred. A convenience sampling method was used. The study population of interest consisted of adult Omani patients infected with various HCV genotypes and treated at either of the two centers with different DAA regimens, with or without RBV, during the study period.

### 2.2. Inclusion and Exclusion Criteria

All adult patients diagnosed with chronic HCV of any genotype who received treatment involving any DAAs were considered eligible for inclusion in the study, regardless of their previous treatment history. Patients co-infected with chronic hepatitis B virus (HBV) and human immunodeficiency virus (HIV) were included. No patients with HCV co-infected with tuberculosis were included. None of the patients included in this study had organ transplantation such as kidney, liver, heart, or lung transplant. A diagnosis of HCV infection was based on anti-HCV positivity for more than six months, along with detectable serum HCV RNA levels using the Abbott RealTime HCV assay (Abbott Molecular Inc., Wiesbaden, Germany) or the COBAS^®^ AmpliPrep/COBAS^®^ TaqMan^®^ (version 2.0) assay, version 2 (Roche Diagnostics, Branchburg, NJ, USA). The lower limits of detection for each assay were 12 and 15 IU/mL, respectively. Patients who were non-compliant with follow-up and those with missing data were excluded from the analysis.

### 2.3. Follow-Up and Primary Outcome

Patients were followed up for a minimum of 6 months after the completion of therapy. Follow-up and surveillance for HCC continued for patients with liver cirrhosis, defined as stage 4, using the METAVIR scoring system or a liver stiffness measurement (LSM) of more than 12.5 kPa using transient elastography. Treatment efficacy was defined as the patient achieving SVR at 12 weeks post-treatment completion (SVR-12) and 24 weeks post-treatment completion (SVR-24), as indicated by undetectable levels of HCV RNA at the respective endpoint.

### 2.4. Data Collection Process

Data were retrieved from the computerized information systems of the two hospitals, as well as through patient interviews during hospital visits. Demographic information, including age, gender, relevant medical history, history of previous HCV treatment, and self-reported alcohol consumption, was recorded for each patient. Baseline physical examinations were conducted during each patient’s initial visit, after 12 weeks of treatment, at the end of treatment, and 12 weeks post-treatment unless otherwise indicated. All adverse events, defined as any new symptoms occurring during the treatment period, reported by patients or their treating physician were recorded. Various blood investigations were conducted prior to treatment initiation, including platelet count, coagulation profile, liver chemistry, electrolyte levels, renal function, hepatitis B virus (HBV) and HCV serology, HCV RNA and HBV DNA in positive cases, HCV genotype, alpha-1 antitrypsin level, serum ceruloplasmin level, iron profile and serum ferritin level, and antinuclear antibody and autoimmune profile.

The Chronic Kidney Disease-Epidemiology Collaboration equation was used to calculate the estimated glomerular filtration rate [19], and chronic kidney disease was staged according to the Kidney Disease: Improving Global Outcomes guidelines [20]. A full blood count, liver chemistry panel, electrolyte assessment, renal profile, and HCV RNA quantification were performed at weeks 4 and 12 of treatment, at the end of treatment, and at 12 and 24 weeks following treatment completion. Additionally, abdominal imaging, including ultrasonography, computed tomography, and/or magnetic resonance imaging, was conducted. The degree of liver fibrosis was assessed using non-invasive means, namely Aspartate Aminotransferase (AST)-to-Platelet Ratio Index (APRI) and Fibrosis-4 Index (FIB-4) scores, as well as liver elastography and in a few cases, liver biopsy. APRI scores of 1 and 2 and FIB-4 scores of 1.45 and 3.25 were considered the lowest and highest cut-off values for advanced fibrosis, respectively [21,22].

Liver stiffness was evaluated using transient elastography (FibroScan^®^, Echosens, Paris, France) at AFH by two experienced hepatology nurses and two-dimensional shear-wave elastography (GE HealthCare Technologies Inc., Chicago, IL, USA) at SQUH by an experienced radiology technician. Cirrhosis was defined as either a liver elastography score of more than 12.5 kPa or based on the clinician’s determination of the patient as cirrhotic in the medical records. In cases where there was discordance between the transient elastography score and clinician assessment, the clinician’s evaluation was considered definitive. All included investigations were required for HCV treatment as per international guidelines, and no extra investigations were performed solely for research purposes.

### 2.5. Treatment Protocol

Treatment decisions were made at the clinician’s discretion, guided by medication availability and the guidelines of the American Association for the Study of Liver Diseases and the European Association for the Study of Liver Diseases [9,10]. Most DAA regimens included an NS5B inhibitor (i.e., SOF) as the backbone of treatment, with the addition of an NS5A inhibitor (e.g., DCV, LDV, elbasvir [ELB], or VEL) or an NS5A inhibitor combined with an NSS3/4A inhibitor (i.e., ELB/grazoprevir [GZR]). The choice of regimen was tailored according to the patient’s HCV genotype, prior HCV treatment history, degree of fibrosis, presence or absence of renal failure, and the overall availability of medications and the availability of combined medications in one tablet. Nearly all second-generation DAAs were available, except for ritonavir-boosted paritaprevir, ombitasvir, and dasabuvir. However, newer regimens such as GLE/PIB and the triple combination of SOF/VEL/voxilaprevir were not yet available.

For difficult-to-treat patients, such as those with a history of prior DAA treatment failure, HCV genotype 3, or decompensated cirrhosis, RBV was added to the regimen. The daily dose of RBV was based on body weight at 800 or 1000 mg for patients weighing < 65 or 65–85 kg, respectively, increasing to 1200 mg in two divided doses for patients weighing > 85 kg. Patients with decompensated cirrhosis received a lower initial RBV dose (600 mg per day), titrated based on tolerance and side effects. No unproved trial medications were used, and all treatment plans were discussed with patients and were based on the published guidelines mentioned above.

### 2.6. Statistical Analysis

Data were analyzed using STATA software, version 14.2 (Stata Corp LLC, College Station, TX, USA). Continuous variables were expressed as means (±standard deviation) or medians (interquartile range [IQR]), while categorical variables were presented as frequencies and percentages. Proportions among patient subgroups were compared using either a Chi-squared test or Fisher’s exact test, as appropriate. Changes in laboratory values from baseline to the SVR-12 time point were compared using either a paired *t*-test or Wilcoxon matched-pairs signed-ranks test, as appropriate. A two-sided *p*-value of <0.05 was considered statistically significant. Ethical approval for this study was obtained from the respective ethics committees of both MCMSS and SQUH.

## 3. Results

### 3.1. Patient Characteristics

A total of 375 patients were included in the analysis. The mean age was 47.3 ± 15.4 years. There was a slightly higher preponderance of male patients (n = 222; 59.2%). The majority of patients had HCV genotype 1 (n = 154; 41.1%), followed by genotype 3 (n = 149; 39.7%). There were no patients with genotypes 5 or 6. Ultrasonography performed within 6 months of treatment initiation revealed liver cirrhosis in 19.7% of patients. Comorbidities such as diabetes and hypertension were present in 24.5% and 27.2% of patients, respectively. Other baseline demographic and clinical characteristics are shown in Table 1.

### 3.2. Treatment Regimens

**Figure 1:**
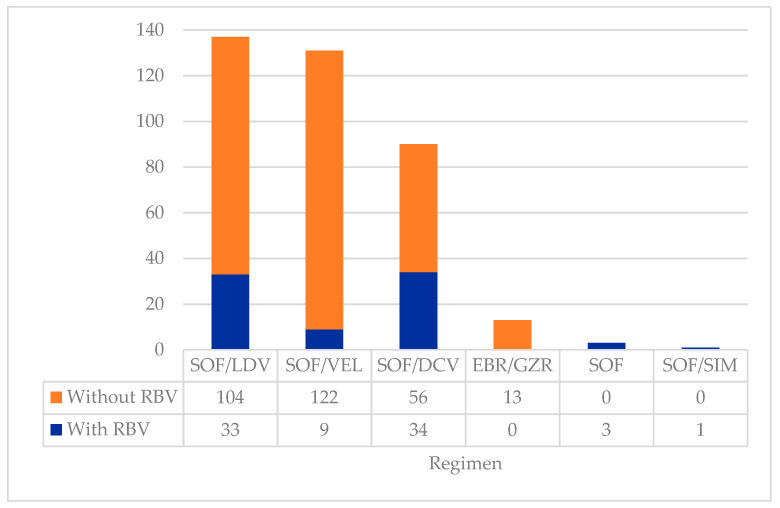
Types of DAA regimens prescribed to the cohort (n = 375). Abbreviations: DAA—direct-acting antiviral; DCV—daclatasvir; EBR—elbasvir; GZR—grazoprevir; LDV—ledipasvir; RBV—ribavirin; SIM—simeprevir; SOF—sofosbuvir; VEL—velpatasvir.

Different DAA regimens were prescribed depending on the patient’s profile, drug interactions, and drug availability. Most patients underwent a 12-week treatment regimen (n = 317; 84.5%), although a minority received treatment for 24 weeks (n = 58; 15.5%). Branded medications were provided to 24 patients (6.4%), with the vast majority receiving generic forms of the medications (n = 351; 93.6%). Figure 1 shows the different DAA regimens prescribed. Patients were most frequently treated with SOF/LDV (n = 137; 36.5%), followed by SOF/VEL (n = 131; 34.9%), with or without RBV. Overall, RBV was added to the DAA regimens of 80 patients (21.3%) of different HCV genotypes, including patients with genotype 1 (n = 27; 7.3%), genotype 2 (n = 3; 0.8%), genotype 3 (n = 35; 9.3%), genotype 4 (n = 14; 3.7%), and mixed genotype (n = 1; 0.3%).

The majority of patients were treatment-naïve (n = 269; 71.7%), although 106 (28.3%) had previously undergone HCV treatment (i.e., were treatment-experienced). Among the latter group, 72 patients (19.3%) had been exposed to pegylated interferon and RBV. Failure with first-generation DAAs (boceprevir or telaprevir) was reported in 13 patients (3.5%), while 21 patients (5.6%) experienced treatment failure with second-generation DAAs, including SOF/DCV (n = 8; 2.1%), SOF/telaprevir (n = 5; 1.3%), SOF/VEL (n = 5; 1.3%), SOF with RBV (n = 2; 0.5%), and SOF with RBV and pegylated interferon (n = 1; 0.3%).

### 3.3. Sustained Virologic Response

**Figure 2:**
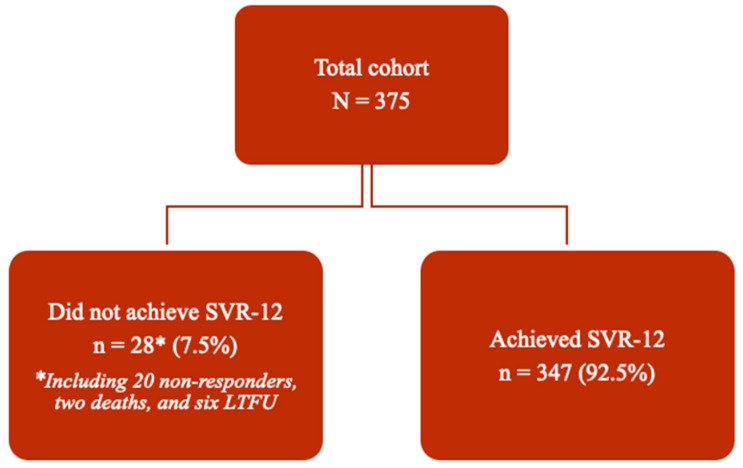
Flowchart depicting outcomes of DAA treatment in the cohort (n = 375). Abbreviations: SVR—sustained virologic response; SVR-12—SVR at 12 weeks post-treatment completion; LTFU—lost to follow-up.

Overall, SVR-12 was achieved successfully by 347 patients, resulting in a treatment efficacy rate of 92.3% by intention-to-treat analysis or 94.6% per protocol analysis. Figure 2 depicts a flowchart of patient outcomes.

Among the subset of 367 patients who completed the treatment protocol, similar SVR-12 rates were observed between genotype 1-infected individuals treated with SOF/LDV and those treated with SOF/VEL (92.3% versus 93.1%). High SVR-12 rates ranging from 93.3 to 100% were seen among difficult-to-treat patients with genotype 3, regardless of treatment regimen. Lower response rates were recorded among patients with different genotypes receiving EBR/GZR regimens (75.0–100%); however, as the number of patients treated with this particular regimen was low, no conclusions could be made. Different DAA regimens and their SVR-12 rates in relation to HCV genotypes are shown in Table 2. No significant differences in SVR-12 rates were noted in terms of either specific treatment regimen (*p* = 0.810) or HCV genotype (*p* = 0.590).

A lower SVR-12 rate was observed among patients with liver cirrhosis at baseline compared to patients without cirrhosis (87.7% vs. 96.3%, *p* = 0.004). In addition, patients with HCC exhibited a significantly lower rate of SVR-12 compared to patients without HCC at baseline (78.6% vs. 95.0%; *p* = 0.009). Other factors such as age or prior treatment experience were not associated with significant differences in SVR-12 rate, nor was the addition of RBV to the regimen or prolongation of treatment from 12 to 24 weeks. Table 3 compares SVR-12 rates across various demographic, clinical, and treatment-related characteristics. Both univariate and multivariate analyses are included, showing the likelihood of failure to achieve SVR-12

### 3.4. Treatment Failure

In total, six patients were lost to follow-up, including one during the second week of treatment, one during the eighth week of treatment, one during the 12th week of treatment week, and three at the SVR-12 visit. In addition, two deaths occurred during the study period, both deemed unrelated to treatment. A total of 20 patients (5.3%) did not achieve SVR-12 due to HCV relapse, indicating treatment failure. The characteristics of these patients are shown in Table 4. Only seven patients (1.9%) failed to achieve SVR-12 twice [see Table A1 in Appendix A].

### 3.5. Post-Treatment Changes

Among the 347 patients who achieved SVR-12 successfully, significant improvements in baseline hepatic necroinflammatory markers, liver function indices, and non-invasive markers of liver fibrosis were observed at 12 weeks post-HCV treatment. In particular, there was a significant decrease in alanine aminotransferase (mean: 81.14 ± 76.03 vs. 23.67 ± 13.35 U/L), AST (mean: 62.36 ± 48.67 vs. 23.67 ± 13.36 IU/L), and total bilirubin (median (IQR): 10 (7, 15) vs. 8 (6, 13) μmol/L) levels (*p* < 0.001 each). We also observed a significant improvement in albumin levels (mean: 41.51 ± 6.22 vs. 43.05 ± 5.51 g/L; *p* < 0.001). In turn, there were statistically significant reductions in non-invasive markers of liver fibrosis (i.e., FIB-4 and APRI scores) as well as LSM (14.11 ± 13.31 vs. 10.54 ± 10.17 kPa) at the SVR-12 timepoint (*p* < 0.001 each). Although there was a slight improvement in the international normalized ratio, this was not statistically significant and was most likely due to the short duration of follow-up. Table 5 details all post-treatment changes in laboratory findings, non-invasive liver fibrosis markers, and liver elastography measurements compared to baseline.

### 3.6. Tolerability of Medications and Adverse Side-Effects

The majority of the patients tolerated the medications very well. The most common adverse events were a non-specific cough (n = 2; 0.5%), fatigue (n = 2; 0.5%), a mild skin rash (n = 1; 0.3%), headache (n = 1; 0.3%), and conjunctivitis (n = 1; 0.3%). At baseline, grade I anemia (defined as hemoglobin [Hb] levels of <10 g/L) was present in 86 patients (22.9%), while 25 (6.7%) had grade II (defined as Hb levels of 8–10 g/L) and seven (1.9%) had grade III anemia (defined as Hb levels of <8 g/L). All patients with baseline anemia exhibited a non-significant improvement in their hemoglobin levels by 12 weeks post-treatment.

## 4. Discussion

The present study aims to provide a comprehensive evaluation of the effectiveness and safety of DAA treatment, drawing on our real-world experience treating chronic HCV infection at two tertiary centers in Oman over a 9-year period. In our study cohort, treatment-naïve patients achieved an SVR-12 rate of 95.0% using various DAA regimens. Similarly, high SVR-12 rates, exceeding 97%, have been documented in multiple cohorts from the Middle East and other regions [4,23,24,25,26,27,28] [see Table A2 in Appendix A].

Among patients who had previously experienced treatment failure with DAAs in our study, the SVR-12 rate was 93.0%. Other studies have reported SVR rates of 95–97% in treatment-experienced patients [29,30]. Seven out of twenty patients in our study who experienced treatment failure had previously failed to respond to DAAs; however, there was no significant difference in the SVR-12 rate based on treatment history. Ahmed et al. [29], Graf et al. [31], and Nabulsi et al. [32] similarly found no statistically significant association between treatment failure and prior DAA treatment experience, aligning with our findings. However, Jain et al. [33] noted a higher frequency of treatment failure among patients with a history of prior treatment. Such differences in findings may be influenced by varying factors such as sample size, host characteristics, viral factors, and treatment regimen.

Liver cirrhosis remains a significant predictor of DAA treatment failure, as evidenced by numerous real-world studies focusing on HCV treatment [30,32,33,34,35]. Within our study cohort, 19.7% of patients presented with liver cirrhosis; these patients exhibited a markedly lower SVR-12 compared to their non-cirrhotic counterparts (87.7% vs. 96.3%). This discrepancy is presumed to stem from the impact of cirrhosis on therapy efficacy and drug metabolism [32]. Although the analysis of resistance-associated substitution (RAS) variants was omitted in our cohort as such testing is not routinely incorporated into our clinical practice protocols, an investigation by Starace et al. [36] identified a high prevalence of RAS mutations among cirrhotic patients who experienced DAA treatment failure in a small Italian cohort.

A statistically significant correlation between HCC and treatment failure was observed within our cohort, mirroring findings across multiple studies [31,34,36,37,38,39]. Research indicates that HCC patients who undergo early intervention for their condition, particularly when tumors are inactive, tend to achieve higher SVR-12 rates [34,36]. However, the precise etiology behind the low SVR-12 rates seen in active HCC cases remains obscure. Plausible hypotheses suggest that HCC may act as a reservoir for HCV replication, potentially leading to hepatic architectural alterations, impaired drug delivery, or the development of resistant HCV strains [36,37,38,39]. Furthermore, a recent study highlighted an elevated prevalence of RAS mutations within tumoral tissues isolated from HCC-affected livers and liver explants, even in the absence of detectable mutations in corresponding plasma samples [38].

Genotype 1 is the most common genotype in our cohort, representing 41.1% of the entire cohort. Notably, this genotype also accounted for the largest proportion of patients with advanced liver disease, including those with cirrhosis and hepatocellular carcinoma (HCC). As described earlier, both cirrhosis and HCC have been identified as significant predictors of lower SVR, which likely explains the reduced treatment success observed in patients with genotype 1 [32,34,36,37,38,39].

Clinical trials have demonstrated that both the addition of RBV to the treatment regimen and extending the overall duration of treatment can enhance sustained SVR rates within distinct patient cohorts, including patients with genotypes 1a or 3, those with cirrhosis, or those with prior treatment experience [31,39]. Nevertheless, within our cohort, neither the administration of RBV nor the prolongation of treatment from 12 to 24 weeks was found to significantly influence treatment response, paralleling outcomes observed in comparable large-scale, retrospective investigations [4,40,41]. This incongruity may stem from the non-systematic or ad hoc nature of RBV incorporation and treatment duration extensions in clinical practice, guided on a case-by-case basis by treatment protocols and clinical assessments targeting specific indications. Further investigation is therefore necessary to evaluate the effect of these variables on rates of virologic response across real-world cohorts.

In our study population, just over one-third (39.7%) of patients had genotype 3, which has been reported as a predictor of treatment failure [34]. However, we observed no statistically significant association between the HCV genotype and the SVR-12 rate. Research from the Saudi population suggests that having a mixed HCV genotype could predict treatment failure [23,42]. Nevertheless, this finding did not align with our cohort, in which all patients with mixed HCV genotypes successfully achieved SVR-12. However, it should be noted that research on mixed HCV genotypes is limited due to their rare occurrence.

Statistically significant improvements in liver necro-inflammatory markers and liver function parameters were observed post-SVR-12 in our cohort. Additionally, compared to baseline measurements, there was a significant reduction in non-invasive indicators of liver fibrosis, including FIB-4, APRI and LSM. These results are consistent with those reported in global studies [42,43,44,45,46]. This improvement may reflect a potential regression of liver fibrosis or an improvement in necroinflammation post-treatment [43]. Further longitudinal studies with a longer follow-up duration are recommended for identifying predictors of fibrosis progression. Although a liver biopsy is more accurate for assessing liver fibrosis post-SVR, it is an invasive procedure carrying the risk of complications. In terms of side effects, our study demonstrated that DAA regimens have excellent efficacy and tolerability, with most adverse effects being mild. None of our patients discontinued treatment due to drug-related side effects, which is consistent with findings from other studies regarding DAA safety and tolerability [44,46,47].

A major limitation of our study was the short duration of follow-up. Even after achieving SVR-12, fibrosis regression can occur gradually, necessitating longer-term studies to accurately assess fibrotic changes. Additionally, this study was retrospective, and various treatment regimens were evaluated with unequal patient distribution between them. Also, this treatment heterogenicity may introduce variability in treatment outcomes. Furthermore, eight patients (2.13%) were excluded from the intention-to-treat analysis due to loss to follow-up; however, this small percentage would be unlikely to significantly influence treatment response rates. Finally, there may be an under-reporting of treatment side effects as our study relied on data collected during routine clinical practice.

## 5. Conclusions

This real-world study underscores the high efficacy and safety of DAA treatment regimens across various HCV genotypes, consistent with current practice guidelines and previous clinical trial findings. Furthermore, our findings indicate post-treatment improvements in hepatic necro-inflammatory and functional markers, along with non-invasive liver fibrosis panel results. Future research should focus on establishing robust tools for assessing changes in liver fibrosis with DAA use to corroborate our results.

## Figures and Tables

**Table 1:** Baseline clinical and demographic data of the cohort (n = 375).

Characteristic	n (%)
Age (years)	
Mean ± SD	47.3 ± 15.4
Gender	
Male	222 (59.2)
Female	153 (40.8)
BMI (kg/m^2^)	
Mean ± SD	26.5 ± 5.9
Comorbidities	
Diabetes	92 (24.5)
HTN	102 (27.2)
SCD	3 (0.8)
Thalassemia	13 (3.5)
HIV co-infection	6 (1.6)
Viral parameters	
HBsAg positivity	14 (3.7)
Anti-HBc isolation	86 (22.9)
Mean HCV RNA level * (logs) ± SD	5.91 ± 0.99
HCV genotype	
1	154 (41.1)
2	6 (1.6)
3	149 (39.7)
4	52 (13.9)
Mixed	4 (1.1)
Unknown	10 (2.8)
HCV-related complications	
Liver cirrhosis †	74 (19.7)
HCC	15 (4.0)

Abbreviations: Anti-HBc—hepatitis B core antibody; BMI—body mass index; HBsAg—hepatitis B surface antigen; HCC—hepatocellular carcinoma; HCV—hepatitis C virus; HIV—human immunodeficiency virus; HTN—hypertension; SCD—sickle cell disease; SD—standard deviation. * Using a polymerase chain reaction. † According to ultrasonography.

**Table 2:** Rates of SVR-12 across different DAA treatment regimens and HCV genotypes (n = 367) *.

Regimen	Frequency of SVR-12, n (%)						Total	*p* Value
	Genotype 1 (n = 150)	Genotype 2 (n = 6)	Genotype 3 (n = 149)	Genotype 4 (n = 50)	Mixed Genotype (n = 4)	Unknown Genotype (n = 8)		
**SOF/DCV**								0.810
With RBV (n = 34)	3 (100)	-	28 (93.3)	1 (100)	-	**-**	32 (94.1)	
Without RBV (n = 55)	4 (100)	-	47 (95.9)	1 (100)	1 (100)	-	53 (96.4)	
**SOF/LDV**					-			
With RBV (n = 33)	21 (95.5)	-	-	11 (100)	-	-	32 (97.0)	
Without RBV (n = 102)	67 (93.1)	0 (0)	3 (100)	23 (100)	3 (100)	-	96 (94.1)	
**SOF/VEL**								
With RBV (n = 9)	1 (100)	2 (100)	4 (100)	1 (100)	-	1 (100)	9 (100)	
Without RBV (n = 118)	37 (92.3)	2 (100)	57 (93.4)	8 (100)	-	7 (100)	111 (94.1)	
**SOF with RBV** (n = 3)	1 (100)	1 (100)	1 (100)	-	-	-	3 (100)	
**EBR/GZR** (n = 12)	6 (85.7)	-	1 (100)	3 (75.0)	-		10 (83.3)	
**SOF/SIM with RBV** (n = 1)	-	-	-	1 (100)	-	-	1 (100)	
Total	140 (93.3)	5 (83.3)	141 (94.6)	49 (98.0)	4 (100)	8 (100)	347 (94.6)	
*p*-value	0.590							

Abbreviations: DCV—daclatasvir; EBR—elbasvir; GZR—grazoprevir; LDV—ledipasvir; RBV—ribavirin; SIM—simeprevir; SOF—sofosbuvir; SVR-12—SVR at 12 weeks post-treatment completion; SVR—sustained virologic response; VEL—velpatasvir. * Using per-protocol analysis (eight patients did not complete treatment). Note: The main treatment regimens are written in bold.

**Table 3:** Rates of SVR-12: Univariate and multivariate analysis based on selected demographic and clinical characteristics, treatment regimen, and treatment duration (n = 367) *.

Variable	Frequency of SVR-12, n (%)	*p*-Value	Univariate	*p* Value	Multivariate	*p* Value
			OR (95%CI) ^‡^		OR (95%CI) ^‡^	
Age (years) <50 (n = 205) >50 (n = 162)	195 (95.1) 152 (93.8)	0.590	1.28 (0.52–3.16)	0.59	1.10 (0.36–0.39)	0.86
Gender Male (n = 217) Female (n = 150)	203 (93.5) 144 (96.0)	0.310	1.66 (0.62–4.41)	0.31	2.16 (0.70–6.66)	0.18
Treatment status Naïve (n = 261) Experienced (n = 106)	248 (95.0) 99 (93.4)	0.540	1.35 (0.52–3.48)	0.54	1.59 (0.56–4.54)	0.39
Treatment duration 12 w (n = 309) 24 w (n = 58)	292 (94.5) 55 (94.8)	0.920	0.94 (0.27–3.31)	0.92	0.72 (0.17–2.96)	0.644
Addition of RBV Yes (n = 76) No (n = 291)	73 (96.1) 274 (94.2)	0.520	0.66 (0.19–2.32)	0.52	0.54 (0.14–2.13)	0.38
Liver cirrhosis Yes (n = 73) No (n = 294)	64 (87.7) 283 (96.3)	0.004 ^†^	3.62 (1.43–9.09)	0.006	3.59 (1.27–10.16)	0.02 ^†^
HCC Yes (n = 14) No (n = 339)	11 (78.6) 322 (95.0)	0.009 ^†^	5.17 (1.32–20.26)	0.02	4.69 (0.90–24.41)	0.066 ^†^
HCV RNA <800,000 (n = 145) >800,000 (n = 218)	137 (94.5) 206 (94.5)	0.990	0.99 (0.40–2.50)	0.99	1.16 (0.43–3.13)	0.764

Abbreviations: HCC—hepatocellular carcinoma; HCV RNA—hepatitis C virus ribonucleic acid; HCV—hepatitis C virus; OR—odds ratio; RBV—ribavirin; SVR-12—SVR at 12 weeks post-treatment completion; SVR—sustained virologic response. * Using per-protocol analysis (eight patients did not complete treatment). ^†^ Statistically significant at *p* < 0.05. ^‡^ Odds ratios represent the likelihood of failure to achieve SVR-12.

**Table 4:** Characteristics of patients who failed to achieve SVR-12 (n = 20).

Characteristic	n (%)
Age (years)	
Mean ± SD	48.1 ± 18.3
Gender	
Male	14 (70.0)
Female	6 (30.0)
HCV genotype	
1	10 (50.0)
2	1 (5.0)
3	8 (40.0)
4	1 (5.0)
Liver cirrhosis	
Yes	9 (45.0)
No	11 (55.0)
Treatment status	
Naïve	13 (65.0)
Experienced	7 (35.0)
Treatment duration (weeks)	
12	17 (85.0)
24	3 (15.0)
Treatment regimen	
SOF/DCV without RBV	2 (10.0)
SOF/DCV with RBV	2 (10.0)
SOF/LDV without RBV	6 (30.0)
SOF/LDV with RBV	1 (5.0)
SOF/VEL without RBV	7 (35.0)
EBR/GZR	2 (10.0)

Abbreviations: DCV—daclatasvir; EBR—elbasvir; GZR—grazoprevir; HCV—hepatitis C virus; LDV—ledipasvir; RBV—ribavirin; SD—standard deviation; SOF—sofosbuvir; SVR-12—SVR at 12 weeks post-treatment completion; SVR—sustained virologic response; VEL—velpatasvir.

**Table 5:** Changes in baseline laboratory values at SVR-12 (n = 347).

Parameter	Mean ± SD		*p* Value
	Baseline	At SVR-12	
Total bilirubin (μmol/L), median (IQR)	10 (7, 15)	8.2 (6, 13)	<0.001 *
ALT ^†^ (IU/L)	81.14 ± 76.03	23.67 ± 13.35	<0.001 *
AST ^†^ (IU/L)	62.36 ± 48.67	23.67 ± 13.36	<0.001 *
Albumin (g/dL)	41.64 ± 6.20	42.99 ± 5.55	<0.001 *
WBC (×10^3^/mm^3^)	5.48 ± 2.90	5.58 ± 4.15	0.330
Hb (g/dL)	12.81 ± 2	12.62 ± 2.08	0.050
Platelet count (×10^3^/mm^3^)	221 ± 103	211 ± 92	0.500
INR	1.22 ± 1.30	1.06 ± 0.16	0.260
FIB-4	2.44 ± 2.80	1.70 ± 1.67	<0.001 *
APRI	1.05 ± 1.31	0.36 ± 0.42	<0.001 *
LSM (kPa)	12.12 ± 10.74	9.82 ± 7.06	<0.001 *

Abbreviations: ALT—alanine aminotransferase; APRI—AST-to-Platelet Ratio Index; AST—aspartate aminotransferase; FIB-4—Fibrosis-4 Index; Hb—hemoglobin; INR—international normalized ratio; IQR—interquartile range; LSM—liver stiffness measurement; SD—standard deviation; WBC—white blood cell count. * Statistically significant at *p* < 0.05. ^†^ Upper limit of normal: 40 IU/L.

## Data Availability

All data generated or analyzed during this study are included in this article. Further inquiries can be directed to the corresponding author.

## Appendix A

**Table A1:** Treatment regimens prescribed to patients who failed to achieve SVR-12 twice (n = 7).

Patient No.	First Regimen	Second Regimen
1.	SOF/LDV with RBV	SOF/VEL
2.	SOF/LDV with RBV	SOF/DCV with RBV
3.	SOF/VEL	SOF/VEL
4.	SOF/DCV with RBV	SOF/VEL
5.	SOF/VEL with RBV	SOF/VEL
6.	SOF/VEL with RBV	SOF/VEL
7.	SOF/DCV with RBV	SOF/VEL

Abbreviations: DCV—daclatasvir; LDV—ledipasvir; RBV—ribavirin; SOF—sofosbuvir; SVR-12—SVR at 12 weeks post-treatment completion; SVR—sustained virologic response; VEL—velpatasvir.

**Table A2:** Direct-Acting Antivirals for the Treatment of Hepatitis C in Oman in comparison to the world.

Region	Genotype	DAAs	Clinical Response (SVR-12)	Outcomes	Ref.
Asia	G-1	SOF/LDV SOF/LDV + RBV	96.2% 100%	- Good safety profile. - Improvement in MELD score in decompensated patients	[4]
	G-3	SOF/DCV SOF/VEL SOF/LDV	97.4% 92.9% 87.5%		
Europe (UK)	G-1	SOF/LDV ± RBV	93%	Predictors of treatment failure: - Male Gender - Genotype 3 - Prior treatment - Cirrhosis Treatment regimen did not significantly impact SVR-12 rates. Treatment relapses were significantly higher in genotype 3 compared to genotype 1	[24]
	G-3	SOF/DCV + RBV	87%		
Africa	G-1	SOF/LDV	96.2%	- Six participants failed treatment, experienced HCV relapse and had resistance mutations - Mild adverse events.	[25]
	G-3	Not reported			
North America (Canada)	G-1	SOF/LDV PTVr/OBV/DSV ± RBV	93% 100%	Predictors of treatment failure: - Older age - Male gender - HCC No significant differences in SVR rates based on treatment centers, HIV coinfection, or liver transplantation	[26]
	G-3	SOF/LDV ± RBV	100%		
South America: Brazil	G-1	SOF/LDV SOF/DCV PTVr/OBV/DSV ± RBV	100% 90% 100%	Predictors of treatment failure: - Cirrhosis - Genotypes 1a or 3 No adverse events were attributed to the DAA therapy	[27]
	G-3	SOF/DCV	93%		
Australia	G1	SOF/LDV SOF/DCV	98% 100%	- 14.2% of patients lost to follow-up - 3.6% experienced virological failure - 0.3% discontinued due to adverse effects - 0.5% discontinued due to noncompliant - 0.7% died during the treatment or follow-up period	[28]
	G3	SOF/DCV	92.4%		

Abbreviations: DCV—Daclatasvir; DAA—Direct-acting antivirals; G-1—Genotype 1; G-3—Genotype 3; HCC—Hepatocellular carcinoma; HCV—Hepatitis C virus; LDV—Ledipasvir; MELD—Model of End-stage Liver Disease; PTVr/OBV/DSV—Paritaprevir/Ombitasvir/Dasabuvir; RBV—Ribavirin; SOF—Sofosbuvir; SVR—Sustained virologic response; SVR-12—SVR at 12 weeks post-treatment completion; VEL—Velpatasvir.
